# Left Atrial Appendage Occlusion vs. Oral Anticoagulants: Current Evidence and Debates

**DOI:** 10.3390/jcm15187015

**Published:** 2026-09-10

**Authors:** Emir Baskovski, Halil Gulyigit, Omer Akyurek, Timucin Altin, Eralp Tutar

**Affiliations:** 1 Department of Cardiology, Ankara University School of Medicine, Ankara 06230, Türkiye; emirbaskovski@gmail.com (E.B.); drakyurek52@gmail.com (O.A.); timucinaltin@gmail.com (T.A.); eralptutar@gmail.com (E.T.); 2 Department of Cardiology, Izmir Tepecik Training and Research Hospital, Izmir 35020, Türkiye

**Keywords:** antithrombotic therapy, atrial fibrillation, device-related thrombus, direct oral anticoagulants, left atrial appendage occlusion, peridevice leak, stroke prevention

## Abstract

Atrial fibrillation (AF) is a major cause of cardioembolic stroke, and oral anticoagulation (OAC) remains the standard of care for thromboembolic prophylaxis. Because the left atrial appendage (LAA) is the dominant source of thrombus in AF, percutaneous LAA occlusion (LAAO) has been developed as a mechanical alternative for patients in whom long-term anticoagulation is problematic. Randomized trials against warfarin established non-inferiority for the composite endpoint, though not consistently across coprimary endpoints, and more recent trials against direct oral anticoagulants (DOACs) have extended this comparison to post-ablation and lower-risk populations. Across these studies a consistent pattern emerges: the clinical advantage of LAAO derives largely from a reduction in non-procedural bleeding rather than from superior ischemic protection, and recent meta-analyses diverge mainly according to how the thromboembolic endpoint is composed. At the extreme of stroke and bleeding risk, LAAO failed to demonstrate non-inferiority to physician-directed medical care. This review synthesizes the randomized evidence, examines methodological differences in endpoint construction and patient selection that complicate cross-trial comparison, and discusses determinants of procedural success including peridevice leak, device endothelialization, and device-related thrombus. Remaining evidence gaps are outlined, particularly for patients who experience ischemic stroke despite adequate anticoagulation.

## 1. Introduction and Objective

Atrial fibrillation (AF) affects nearly 60 million individuals worldwide and exerts a substantial clinical and economic burden on modern healthcare systems due to its high morbidity and mortality rates [1,2].

The onset of AF disrupts the organized mechanical contraction of the left atrium (LA), leading to blood stasis within the chamber. Pathophysiologically, in non-valvular AF the large majority of left atrial thrombi—approximately 90% in the pooled imaging, autopsy and surgical series from which the estimate derives—are confined to the left atrial appendage (LAA), a blind, tortuous, pouch-like structure whose complex anatomy predisposes to flow stagnation. The proportion is considerably lower in rheumatic AF, in which a substantial share of thrombus arises within the atrial cavity itself, and the estimate should not be extended to that population [3]. Endothelial damage, stasis, and hypercoagulability, which are the elements of Virchow’s triad, make the LAA a major source of embolism. The anatomical structure of the LAA is more trabeculated than the LA, and its contractile function is more readily impaired [4]. Embolization of these thrombi into the systemic circulation results in ischemic strokes that typically involve large vascular territories and carry high mortality and morbidity [3]. In current clinical practice, oral anticoagulation, primarily with direct oral anticoagulants (DOACs), is the mainstay of preventing AF-related thromboembolism [2].

Despite their proven efficacy, these agents have important limitations, including the need for lifelong therapy and the associated problems of adherence, an inherent risk of major bleeding, restricted use in hepatic and renal impairment, and drug–drug interactions [5]. Left atrial appendage occlusion (LAAO) was developed as a mechanical alternative to overcome these limitations.

In patients who are already scheduled for cardiac surgery, surgical LAAO carries a Class 1 recommendation in current guidelines [2,6]. This recommendation is based on a randomized controlled trial, the Left Atrial Appendage Occlusion Study III (LAAOS III) [7]. By demonstrating that surgical closure of the appendage in patients with AF undergoing cardiac surgery reduces stroke risk by a further 33%, this study positioned surgical LAAO not as an alternative to anticoagulation therapy but as a synergistic and complementary mechanism that enhances its efficacy [2,6]. Unlike the surgical approach, which guidelines view as a synergistic addition to anticoagulation, percutaneous LAAO strategies have been developed as a potential replacement for long-term oral anticoagulation. They are primarily targeted at patients who cannot tolerate these drugs, those who experience recurrent thromboembolic events despite adequate anticoagulation, or, controversially, those who prefer to avoid long-term anticoagulant therapy [8].

Unlike surgical LAAO, the percutaneous approach introduces an intracardiac device that is inherently thrombogenic, thereby necessitating a period of post-procedural antithrombotic cover. The intensity of that cover is not fixed: regimens range from full-dose anticoagulation through dual or single antiplatelet therapy to, in a minority of patients, no antithrombotic treatment at all, an issue examined in Section 4. Partly for this reason, contemporary literature and meta-analyses remain inconclusive as to whether the intervention provides a net clinical benefit across all patient populations [9]. This lack of definitive evidence may explain why guideline recommendations for percutaneous LAAO remain conditional and divergent. The 2024 ESC guidelines assign a Class IIb, Level C recommendation to percutaneous LAAO in patients with contraindications to long-term anticoagulation, whereas the 2023 ACC/AHA/ACCP/HRS guidelines upgraded the corresponding recommendation to Class 2a, with a further Class 2b recommendation for patients at high bleeding risk on anticoagulation in whom the decision rests on patient preference. Neither reaches the Class 1 level assigned to surgical occlusion performed during cardiac surgery. The 2025 SCAI/HRS guidelines, graded by GRADE methodology rather than by class of recommendation, go further: they suggest LAAO over no therapy where anticoagulation is contraindicated, a conditional recommendation of very low certainty, and present anticoagulation and LAAO as acceptable alternatives for patients who could receive either [8]. The three documents agree on the contraindication group and diverge on how far the indication extends beyond it [2,6,8].

Therefore, the objective of this review is to examine the pathophysiological rationale for percutaneous LAAO and to provide clinicians with an evidence-based roadmap for selecting the ‘right strategy for the right patient’ in a rapidly evolving field.

### Literature Search

This narrative review is based on a structured but non-systematic search of PubMed, performed without date restriction up to July 2026. The search combined the terms “left atrial appendage occlusion” and “left atrial appendage closure”. Priority was given to randomized controlled trials comparing percutaneous LAAO with oral anticoagulation or with best medical therapy, to meta-analyses of those trials, and to current guideline and expert consensus documents. Observational cohorts and registries were included where randomized data were unavailable, in particular for post-procedural antithrombotic management and for patients with thromboembolism despite anticoagulation. Reference lists of retrieved articles were screened for additional sources. Only English-language publications were considered. Because the review was designed to synthesize rather than to pool evidence, no formal risk-of-bias assessment or quantitative synthesis was undertaken.

## 2. Evaluation of Current Studies Conducted on Percutaneous LAAO

The landmark clinical evidence validating the efficacy of mechanical left atrial appendage occlusion has been established through a series of randomized controlled trials. Foremost among these, PROTECT-AF was a pioneering, multicenter, and randomized study comparing the efficacy and safety of the percutaneous LAAO device against warfarin anticoagulation, then the standard therapy, for stroke prevention in patients with AF. A total of 707 patients were randomized in a 2:1 ratio to LAAO device or warfarin arms. The primary composite efficacy endpoint comprised stroke, systemic embolism, and cardiovascular or unexplained death. In initial analysis, the device demonstrated non-inferiority to warfarin therapy; at a mean follow-up of 3.8 years, the LAAO strategy achieved superiority, with significant reductions in all-cause mortality and particularly the risk of hemorrhagic stroke. Although early phases of the PROTECT-AF trial showed higher complication rates—such as pericardial effusion—in the LAAO group, these events declined as operator experience increased. Ultimately, PROTECT-AF became the first randomized trial to demonstrate that mechanical LAA occlusion is a viable alternative to chronic warfarin therapy [8,9,10].

Nevertheless, the high rate of early periprocedural complications in PROTECT-AF highlighted a critical learning curve, demonstrating that the initial safety profile of the device was heavily operator-dependent. The PREVAIL trial was therefore designed as a follow-up study to address these safety concerns, to establish the procedural reproducibility of mechanical occlusion, and to determine whether accumulating operator experience improved safety. This multicenter study randomized 407 patients with AF and again compared the efficacy and procedural safety of device implantation with warfarin. The first coprimary efficacy endpoint at 18 months—a composite of stroke, systemic embolism, and cardiovascular or unexplained death—did not meet the criteria for statistical non-inferiority (rate ratio 1.07; 95% CrI 0.57–1.89). The principal contribution of the trial lies in procedural safety; compared with PROTECT-AF, a marked reduction was recorded in peri-procedural complications such as pericardial effusion, cardiac tamponade, and acute bleeding [11], confirming that advancing operator experience could mitigate the early hazards of the intervention.

While PROTECT-AF and PREVAIL established LAAO as an alternative to warfarin, the widespread adoption of DOACs and catheter ablation marked the beginning of a new era in this field. The OPTION trial randomized 1600 patients who had undergone AF ablation in a 1:1 ratio to percutaneous LAAO with the Watchman FLX device or to oral anticoagulation (95% of whom received a DOAC), with follow-up over 36 months. LAAO was performed 3 to 6 months after ablation in approximately 60% of the patients, and concomitantly with ablation in the remaining 40%. The primary efficacy endpoint (all-cause death, stroke, or systemic embolism) was found to be 5.3% in the LAAO group and 5.8% in the DOAC group (*p* < 0.001 for noninferiority). Non-procedural major bleeding or clinically relevant non-major bleeding occurred in 8.5% of the LAAO group compared with 18.1% of the OAC group (*p* < 0.001 for superiority) [12].

From a methodological standpoint, OPTION trial differs from PROTECT-AF and PREVAIL in one important respect: whereas the earlier trials restricted their mortality component to cardiovascular and unexplained deaths, OPTION incorporated all-cause mortality. The choice is defensible in itself, since all-cause mortality avoids the misclassification inherent in adjudicating cause of death. It nonetheless has a consequence that should be borne in mind when OPTION is set beside trials that counted only cardiovascular death: deaths the intervention cannot plausibly modify accrue in both arms and dilute any true between-group difference, which in a non-inferiority design makes the criterion easier to meet. Furthermore, the OPTION trial focused on a distinct patient population compared to previous studies. The cohort was younger and lower risk with a mean CHA_2_DS_2_-VASc score of 3.2 and a HAS-BLED score of 1.1 which limits the extent to which these findings can be generalized to high-risk AF populations [12].

Long-term adherence to anticoagulation remains difficult even in patients at relatively low-risk, and CHAMPION-AF was designed to test whether percutaneous LAAO could serve as a first-line alternative to DOAC treatment in a broader AF population. Approximately 3000 patients were randomized to Watchman FLX or a DOAC. Event rates for the composite of cardiovascular death, stroke, and systemic embolism at three years were 5.7% with the device and 4.8% with a DOAC an absolute difference of 0.9 percentage points (95% CI −0.8 to 2.6), which satisfied the prespecified non-inferiority margin (*p* < 0.001) [13].

Rates of non-procedural bleeding (the primary safety endpoint), were 10.9% with the device and 19.0% with DOAC, an absolute reduction of 8.1 percentage points (95% CI −10.8 to −5.5) that met the prespecified criterion for superiority (*p* < 0.001). Ischemic stroke was numerically more frequent in the device group than in the DOAC group (3.2% versus 2.0%), although the difference did not reach statistical significance. At three years, life-threatening major bleeding occurred in 1.6% of the LAAO group and 1.8% of the DOAC group, with no significant difference between them [13]. Two points of interpretation follow. First, efficacy and safety were assessed as separate endpoints, so the reduction in non-procedural bleeding does not contribute to, and cannot be said to sustain, the demonstration of non-inferiority for the efficacy composite. That composite was met on its own terms, although the low absolute event rates observed relative to those assumed at the design stage render the conclusion less robust than the *p* value alone implies: applied to the observed comparator rate, the prespecified margin admits a relative risk of 2.0, and the upper bound of the observed hazard ratio (1.20; 95% CI 0.87–1.66) does not exclude a substantial relative increase in events. Second, the bleeding-related component contained within the efficacy composite showed no benefit, since hemorrhagic stroke occurred at identical rates in the two arms (0.4%); the numerical excess is therefore ischemic. What the bleeding reduction does support is the net clinical profile of the strategy, formally captured in the prespecified secondary composite of cardiovascular death, stroke, systemic embolism or non-procedural bleeding, which favored the device (15.1% versus 21.8%; HR 0.66; 95% CI 0.56–0.79). This net clinical benefit should be kept distinct from the formal demonstration of efficacy non-inferiority [14].

Because OPTION and CHAMPION-AF both enrolled patients at low bleeding risk, their findings cannot be extrapolated to the high-bleeding-risk population (the very group in whom treatment poses the greatest challenge). This population had in fact been addressed earlier: PRAGUE-17, a multicenter randomized trial, assigned patients with AF at high risk of both stroke and bleeding to percutaneous LAAO or DOAC therapy.

PRAGUE-17 randomized 402 patients (201 to each group), with apixaban accounting for 95% of DOAC use. The participants were followed up for a median of 3.5 years (totaling 1354 patient-years). The primary composite endpoint comprised stroke, transient ischemic attack, systemic embolism, cardiovascular death, clinically relevant bleeding, and device- or procedure-related complications. This construction differs importantly from the endpoints used in OPTION and CHAMPION-AF, since it incorporates both bleeding and procedural harm within a single measure of net clinical benefit. On this endpoint, LAAO remained non-inferior to DOAC therapy (sHR 0.81; 95% CI 0.56–1.18; *p* = 0.27; *p* = 0.006 for non-inferiority). These findings suggest that in patients at high baseline bleeding risk, LAAO yields net clinical outcomes comparable with those of DOAC therapy once procedural complications are fully accounted for, with a significant reduction in non-procedural bleeding (sHR 0.55; 95% CI 0.31–0.97; *p* = 0.039) [15].

Nonetheless, the clinical balance alters at the extreme of combined ischemic and hemorrhagic risk. CLOSURE-AF enrolled 912 patients selected on exactly those grounds: mean age approximately 78 years, a mean CHA_2_DS_2_-VASc score of 5.2, and a mean HAS-BLED score of 3.0. Despite a high successful implantation rate of 98%, LAAO failed to meet non-inferiority compared with physician-directed best medical care (which for most patients meant DOAC therapy) for the primary composite of stroke, systemic embolism, major bleeding, or cardiovascular or unexplained death (16.8 versus 13.3 events per 100 patient-years; *p* = 0.44 for non-inferiority). Periprocedural complications occurred in 5.7% of patients within 7 days, including major bleeding (4.0%) and pericardial tamponade (1.1%), underscoring that in a population with this risk profile the early procedural burden can offset the long-term benefit of mechanical occlusion. Frailty was not an enrolment criterion in this trial; its findings are therefore evidence about patients at very high combined ischemic and bleeding risk, among whom advanced age is common but not the defining feature [16,17].

A recent meta-analysis pooled six randomized trials comprising 7004 patients. Across these trials, ischemic stroke or systemic embolism occurred more often with LAAO than with OAC 134 events versus 80 (risk ratio [RR] 1.41, 95% confidence interval [CI] 1.07–1.86), rated as moderate certainty of evidence under the Grading of Recommendations, Assessment, Development and Evaluations (GRADE) framework. No corresponding differences emerged for overall stroke or systemic embolism, all-cause mortality, cardiovascular or non-cardiovascular mortality, major bleeding, or hemorrhagic stroke [18]. A second meta-analysis, restricted to four trials of LAAO against DOAC therapy (5890 patients), found no significant differences in all stroke, hemorrhagic stroke, systemic embolism, all-cause mortality, or cardiovascular mortality. Ischemic stroke was numerically more frequent with LAAO (RR 1.36; 95% CI 0.99–1.89; *p* = 0.06), and this difference reached significance when the OPTION trial was excluded (RR 1.45; 95% CI 1.03–2.06; *p* = 0.03). Non-procedural bleeding was substantially reduced with LAAO, whereas overall major bleeding and intracranial hemorrhage did not differ [19]. A third meta-analysis of seven trials (7353 patients) found LAAO comparable to OAC for the composite of stroke or systemic embolism (RR 1.10; 95% CI 0.82–1.48), with no significant differences in ischemic stroke, hemorrhagic stroke, cardiovascular mortality, or all-cause mortality. Clinically significant bleeding was less frequent after LAAO [20]. Read together, these analyses point to a potential ischemic signal rather than to an established difference in ischemic efficacy, and the distinction matters. No individual randomized trial was powered for ischemic stroke as a discrete endpoint; in CHAMPION-AF the excess was numerical and did not reach significance. Across the pooled analyses the direction of effect is consistent but the statistical conclusion is not, and it tracks the construction of the endpoint: significance was reached where ischemic stroke or systemic embolism was analyzed in isolation, was not reached in the DOAC-restricted analysis (*p* = 0.06), and was absent where the composite of all stroke or systemic embolism was used. The single significant estimate in the DOAC-restricted analysis appeared only on sensitivity analysis after exclusion of the OPTION trial, and therefore rests on a modified rather than the primary evidence base. What the aggregate data do support is that LAAO carries mortality comparable to OAC and reduces non-procedural bleeding; whether it affords marginally less ischemic protection remains unresolved. Section 3 considers the mechanisms that would plausibly generate such a signal if it proves real: device-related thrombus (DRT) during the endothelialization window, peridevice leak, and embolic sources outside the appendage.

One randomized trial of appendage intervention sits outside the comparison summarized in Table 1 and is therefore considered separately. It differs from the trials above in technique, in the question posed and in the comparator: it evaluated epicardial ligation rather than endocardial occlusion, tested the intervention as an adjunct to ablation rather than as a substitute for anticoagulation, and used rhythm control rather than thromboembolic protection as its principal endpoint.

Beyond the traditional role of LAAO in stroke prevention and bleeding reduction, clinical investigations have also explored the additional therapeutic effects of interventions such as the LARIAT ligation system on ablation success and rhythm control. The aMAZE trial assessed the safety and efficacy of percutaneous left atrial appendage ligation adjunctively to pulmonary vein isolation (PVI) catheter ablation therapy in patients with non-paroxysmal atrial fibrillation. At 12 months, the freedom from atrial arrhythmias was 64.3% with LAA ligation plus PVI and 59.9% with PVI alone; this 4.3% absolute difference failed to meet the statistical threshold for superiority. Despite demonstrating high occlusion rates post-procedure and satisfying prespecified safety criteria, adjunctive LARIAT ligation therefore conferred no significant rhythm-control advantage over ablation alone, and the technique has not entered routine guideline recommendations. Exploratory subgroup analyses nevertheless suggested possible benefit in patients with early persistent AF and in those with larger left atrial volumes—observations that require prospective confirmation [21].

The trial nonetheless bears on the wider question of whether excluding the appendage confers benefit beyond embolic protection, and its neutral result argues against a clinically meaningful rhythm-control effect from ligation in this setting.

Table 1 summarizes the principal randomized trials of percutaneous LAAO. Baseline patient characteristics differ considerably across these trials, particularly the CHA_2_DS_2_-VASc and HAS-BLED thresholds used for enrollment. This heterogeneity substantially complicates any direct comparison of ischemic stroke prevention and procedural safety across studies.

## 3. Determinants of Procedural Success and Long-Term Outcome

No universally accepted algorithm for patient selection has yet emerged, largely because trial designs and target populations have differed so widely. Several recurring considerations can nonetheless be identified across these studies. Current meta-analyses and subgroup analyses indicate that the success of percutaneous LAAO depends on more than device placement at the appendage orifice: it reflects a multi-component process encompassing post-procedural endothelialization, peridevice leak, and the risk of DRT [8,22,23].

Patient selection turns on the interaction of several related but distinct considerations, and collapsing them onto a single axis of age or frailty obscures more than it clarifies. Percutaneous LAAO front-loads its risk: the procedural hazard is incurred once, whereas the bleeding avoided accrues only over years of anticoagulation not taken. The operative question is therefore whether the expected duration of benefit is long enough to repay that upfront cost, and this depends on life expectancy and competing risks rather than on chronological age as such. Baseline bleeding risk sets the magnitude of the benefit available, since a patient with a low HAS-BLED score has comparatively little bleeding to avoid, as the CHAMPION-AF cohort illustrates, while procedural risk, determined by vascular access, atrial tissue characteristics, appendage anatomy and institutional volume, sets the cost. Current guidance puts a floor under this reasoning, noting that LAAO may be inappropriate where quality life expectancy is less than one year [8]. Clinical vulnerability enters separately again: reduced physiological reserve raises the probability that a periprocedural complication becomes consequential, independently of how long the patient is expected to live. These considerations do not align neatly along an age gradient. A younger patient with an absolute contraindication to anticoagulation and a long horizon of benefit is a strong candidate; so, potentially, is an older patient with recurrent major bleeding, favorable anatomy and preserved functional status. Conversely, a patient of any age in whom competing mortality is high, or in whom the bleeding to be avoided is modest, is a weak one. The available evidence does not support a simple dichotomy between younger and elderly or frail candidates, and CLOSURE-AF should not be read as establishing one; what that trial does show is that at the extreme of combined ischemic and bleeding risk the balance can fall against intervention [8,24].

Bleeding risk must be weighed carefully. Patients with clear contraindications to long-term DOAC have long represented the core target population, providing a strong clinical rationale for percutaneous LAAO procedures [6]. At the other end of the spectrum, patients at low bleeding risk who wish to avoid lifelong oral anticoagulation resemble the CHAMPION-AF cohort, in which LAAO met its non-inferiority criterion and reduced non-procedural bleeding, although without advantage in ischemic stroke prevention [13]. Where left atrial appendage anatomy is unfavorable or institutional experience limited, however, continued DOAC therapy remains the more prudent approach.

Anatomical unsuitability is among the most common reasons for procedural failure. An LAA ostial diameter exceeding the sizing range of available devices, or insufficient appendage depth, prevents full device expansion and predisposes to device embolization or peridevice leak. Such a leak alters local flow within the incompletely occluded pouch, and in the absence of systemic anticoagulation this stasis may promote thrombus formation—a plausible contributor to the numerically higher ischemic stroke rates observed in the device arms of several trials. Implantation technique is a modifiable determinant alongside anatomy. In a dual-centre series of 310 consecutive Watchman FLX procedures, greater device compression than nominal was independently associated with a higher rate of complete appendage occlusion on follow-up transesophageal echocardiography, although the observational design and short follow-up limit what can be concluded [22,25].

Endothelialization of the device surface remains incomplete for weeks to months after implantation; during this interval the exposed frame acts as a prothrombotic substrate and the risk of DRT is greatest. Furthermore, because LAAO addresses a single anatomical source, it offers no protection against non-LAA emboli such as those arising from severe left atrial spontaneous echo contrast or aortic atheroma. The transient hazard of DRT and the residual vulnerability to non-LAA embolic sources together offer a mechanistic explanation for the ischemic signal reported in some LAAO cohorts relative to patients maintained on continuous systemic anticoagulation, should that signal be confirmed [23].

Patients with advanced chronic kidney disease (estimated glomerular filtration rate [eGFR] <30 mL/min) face risk on two fronts. First, contrast administered during the procedure may precipitate acute kidney injury. Second, uremia confers a markedly increased tendency to both bleeding and thrombosis. The available evidence is observational: in the largest long-term series advanced CKD was present in 14% of patients undergoing LAAO and was associated with substantially higher five-year mortality, a difference that plausibly reflects the cardio-renal burden of these patients rather than an effect of the device [26]. A propensity-matched comparison from the same group found no significant difference between LAAO and continued anticoagulation in the primary composite endpoint at three years (22.2% versus 34.2%; HR 0.63, 95% CI 0.35–1.11), in a sample too small to exclude a clinically relevant difference [27].

## 4. Antithrombotic Therapy After Percutaneous LAAO

The rationale for percutaneous LAAO is to release the patient from the cumulative hazard of long-term anticoagulation, yet the implanted device presents a bare, prothrombotic surface to the left atrial circulation until it is covered by neoendothelium. Antithrombotic cover is therefore required precisely in the population selected for its inability to tolerate such therapy. This tension admits no clean resolution, and it explains why post-procedural management remains the least standardized element of the entire LAAO pathway.

The regimen validated in the pivotal trials was derived empirically rather than tested against alternatives. In PROTECT-AF and PREVAIL, patients received warfarin with aspirin for 45 days, were de-escalated to aspirin and clopidogrel until six months if transesophageal echocardiography showed a peridevice leak below 5 mm, and continued aspirin indefinitely [9,10,11]; this schedule was incorporated into the device labeling. Practice has since diverged from that template: subsequent studies replaced warfarin with a DOAC and then dispensed with post-procedural anticoagulation altogether, moving directly to dual antiplatelet therapy [8]. CHAMPION-AF reflects this pluralism, permitting a DOAC with aspirin, a DOAC alone, or dual antiplatelet therapy for three months before de-escalation to antiplatelet monotherapy [13]. What began as a single prescribed protocol has become a menu of permitted options, none of which has been shown superior to another in a randomized comparison.

Real-world practice has drifted further still, and in opposite directions on either side of the Atlantic (Table 2). In the NCDR LAAO Registry, only 12.2% of 31,994 patients received the complete protocol studied in the pivotal trials; discharge on warfarin with aspirin accounted for 36.9%, DOAC with aspirin for 20.8%, and dual antiplatelet therapy for only 5.0% [28]. In the Watchman FLX era the distribution shifted again, with DOAC plus aspirin (48.3%) and DOAC alone (22.6%) predominating among 53,878 patients [29]. European practice inverts these proportions: dual antiplatelet therapy was the discharge regimen in 60% of EWOLUTION participants [30], and in the Italian multi-device registry the 428 patients were distributed almost evenly between dual antiplatelet therapy (27%), single antiplatelet therapy (26%) and oral anticoagulation (27%), with 6% discharged on no antithrombotic treatment whatsoever [31]. The determinant of these choices appears to be the operator’s perception of bleeding risk rather than any trial evidence.

The comparative data that do exist are observational and point, with reasonable consistency, away from combination therapy. In the NCDR analysis, discharge on a DOAC alone carried lower rates of major adverse events (HR 0.78; 95% CI 0.68–0.91) and major bleeding (HR 0.69; 95% CI 0.60–0.80) than DOAC with aspirin, differences that persisted at six months [29]. A network meta-analysis of 41 studies and 12,451 patients likewise placed DOAC monotherapy first for both thromboembolic events and major bleeding, and found dual antiplatelet therapy superior to single antiplatelet therapy for thromboembolic protection without an excess of bleeding [32]. In the Italian registry, oral anticoagulation was associated with better outcomes than dual antiplatelet therapy [31]. These analyses share a decisive limitation: the regimen is selected by the physician on the basis of perceived bleeding risk, so the patients receiving the least intensive therapy are also those at highest hemorrhagic risk, and confounding by indication runs in the direction that would exaggerate the apparent advantage of anticoagulation. Adequately powered randomized comparisons of post-implant antithrombotic regimens are lacking, and current guidance reflects that uncertainty: the 2025 SCAI/HRS panel suggests either postprocedural anticoagulation or dual antiplatelet therapy, a conditional recommendation of low certainty, noting that dual antiplatelet therapy is a reasonable choice where contraindications to anticoagulation are more substantial. It makes no recommendation on single antiplatelet therapy against any other regimen, nor on the optimal duration of treatment, listing both as knowledge gaps [8].

The duration of therapy is tied to a biological process that is neither uniform nor directly observable. Endothelialization is generally held to be complete within 30 to 90 days, but it varies between patients and remains incomplete in a proportion of devices well beyond that window [8]; DRT occurs in approximately 4% of patients within the first year and is concentrated in the early phase [31]. Peridevice leak compounds the problem by maintaining communication between the appendage and the atrium, so that a residual pouch continues to be perfused at low velocity in a patient whose systemic anticoagulation has been withdrawn. This argues for an imaging-guided rather than a calendar-based approach to de-escalation, in which persistence of leak or of an uncovered device surface prolongs therapy. Such an approach is consistent with the framework proposed for transcatheter structural interventions more broadly [33], but it has not been prospectively validated for LAAO.

The net position is uncomfortable. The optimal agent, intensity and duration of post-LAAO antithrombotic therapy are unresolved; the available evidence is observational and confounded; and the patients in whom the question matters most, those with an absolute contraindication to anticoagulation, are systematically under-represented in the datasets that might answer it. Until randomized data are available, the choice remains an individualized judgment weighing residual thrombotic risk against the bleeding profile that prompted referral in the first place.

## 5. Novel Perspectives on Percutaneous LAAO

Moving beyond current guideline recommendations, which are anchored on patients in whom long-term anticoagulation is contraindicated and extend beyond it only conditionally, it is crucial to design clinical trials that encompass new patient populations. For example, no randomized controlled trial evaluating the efficacy and safety of percutaneous LAAO in patients who experience ischemic stroke despite DOAC therapy, and who have no contraindication to DOAC use, has yet been completed. The most substantial evidence in this field comes from the STR-OAC registry [34], a multicenter and retrospective cohort study evaluating percutaneous LAAO efficacy in AF patients who experience ischemic stroke despite being under OAC therapy. In the propensity score-matched analysis with two-year follow-up data, patients who underwent LAAO (n = 433) were compared with patients who continued medical therapy alone (n = 433). The annual recurrent ischemic stroke rate was 2.8% in the LAAO group and 8.9% in the group receiving OAC alone; it revealed that the percutaneous LAAO strategy significantly reduced the stroke risk by 67% (HR: 0.33, 95% CI: 0.19–0.58, *p* < 0.001). The study suggests that LAAO may be a therapeutic option for secondary stroke prophylaxis in this high-risk population in whom OAC fails. These figures require caution. STR-OAC is a retrospective collaboration whose comparator was assembled from separate prospective cohorts rather than from a concurrent control arm, and the two groups differed in the very exposure that defines the population: anticoagulation was adequate before the index event in 92% of the LAAO group and 57% of the controls. A third of the LAAO patients continued anticoagulation afterwards, so the comparison is not cleanly one of replacement. The estimate is hypothesis-generating and does not stand alongside the randomized evidence summarized in Table 1. The ADD-LAAO trial targets the same population but tests LAAO as an adjunct to continued anticoagulation rather than as a replacement. Similarly, the Occlusion-AF trial, which compares percutaneous LAAO with DOAC therapy for secondary stroke prophylaxis, is another randomized study that will strengthen the evidence base [35,36].

Device technology is a second area in which evidence lags practice. The randomized evidence base rests predominantly on a single device family; epicardial ligation has been tested only as an adjunct to ablation and never against anticoagulation for stroke prevention; and designs intended to avoid a permanent implant have not yet reached clinical evaluation. Comparative data across device designs remain limited, and trials addressing these questions are needed before any of these technologies can be positioned in the treatment pathway. A related question is whether neutral results obtained with earlier technology reflect the concept or its execution. Percutaneous epicardial access, for instance, has changed since the ligation trials were designed: pericardial insufflation of carbon dioxide, first described in patients undergoing appendage ligation, was introduced to separate the heart from the parietal pericardium and make subxiphoid needle entry into the empty pericardium safer [37]. Endocardial devices have likewise passed through several iterations. Whether refinements of this kind translate into better clinical outcomes is unknown and can be settled only by trials comparing current-generation technology.

Surveillance after implantation rests on similarly thin evidence. Current guidance suggests follow-up imaging rather than none, in order to detect peridevice leak and device-related thrombus, but grades that recommendation as conditional with very low certainty and identifies the management of a detected leak as an explicit knowledge gap [8]. One of the most notable methodological deficiencies in the LAAO literature is that peridevice leaks are still classified by simple millimetric thresholds (<3 mm or <5 mm) derived from two-dimensional transesophageal echocardiography (2D-TEE). In an era of multislice computed tomography (CCTA) and 4D flow imaging, not only the linear width of a leak, but also the three-dimensional volumetric space it creates and the flow patterns within it, may be the parameters that truly determine clinical outcome. These modalities may help identify the mechanism of cryptogenic stroke after LAAO [38]. Future clinical studies should standardize this assessment.

## 6. Conclusions

Percutaneous LAAO is an established option for a defined group of patients, not a general alternative to anticoagulation. In randomized trials it has met its prespecified non-inferiority criterion against DOACs in high-bleeding-risk, post-ablation and anticoagulation-eligible populations. At the extreme of combined ischemic and bleeding risk it has not. Guidelines agree on its place in patients who cannot take anticoagulants and diverge beyond that point, and nothing in the recent evidence supports its use as a first-line strategy in unselected patients.

Three limitations define the remaining uncertainty. The advantage demonstrated so far comes from reduced non-procedural bleeding, not from better ischemic protection, and a possible ischemic excess remains unresolved. The procedural risk is immediate while the benefit accrues over years, so life expectancy and competing risks weigh as heavily as bleeding risk. And the antithrombotic regimen needed while the device heals has never been tested in an adequately powered randomized comparison, in precisely the patients selected for their inability to tolerate such therapy.

LAAO is therefore an individualized decision rather than a categorical one.

Finally, another critical focus is the “anticoagulant failure” population (patients who experience a stroke despite DOAC therapy). As outlined in the preceding sections, current clinical evidence in this specific subgroup remains limited. However, building on promising retrospective data like the STR-OAC study, ongoing randomized trials such as ADD-LAAO and Occlusion-AF are expected to address this persistent knowledge gap.

## Figures and Tables

**Table 1:** Randomized Trials Comparing Percutaneous LAAO with Anticoagulation or Best Medical Therapy: Design, Outcomes, and Limitations.

Trial (Year)	N	Baseline Stroke Risk Score	HAS-BLED	Comparison	Key Outcomes	Principal Limitations
**PROTECT-AF (2009; 3.8-yr follow-up 2014) [9,10]**	707 (2:1)	CHADS_2_ 2.2–2.3	NR	Watchman vs. warfarin	Non-inferiority met at 18 mo; superiority for the primary composite at 3.8 yr. All-cause mortality 3.2 vs. 4.8 per 100 patient-years (*p* = 0.04); CV mortality 1.0 vs. 2.4 (*p* = 0.005); hemorrhagic stroke 0.2 vs. 1.1. No significant difference in ischemic stroke, an endpoint for which the trial was not powered.	High early periprocedural complication rates, notably pericardial effusion; safety profile heavily operator-dependent. Enrolment threshold (CHADS_2_ ≥ 1) is low by contemporary standards. Comparator no longer reflects standard care.
**PREVAIL (2014) [11]**	407 (2:1)	CHADS_2_ 2.6	NR	Watchman vs. warfarin	First coprimary (18-mo composite of stroke, SE, CV/unexplained death): non-inferiority not met (rate ratio 1.07; 95% CrI 0.57–1.89). Second coprimary (stroke or SE > 7 days): non-inferiority met. Early safety: events in 2.2%, meeting the prespecified goal; broader adverse-event definition 4.2% vs. 8.7% in PROTECT-AF (*p* = 0.004). Implant success 95% (93% among new operators).	Low absolute event rates limited power for the first coprimary endpoint. Warfarin comparator. Confirms procedural learning-curve effects rather than establishing efficacy.
**PRAGUE-17 (2020; 4-yr follow-up 2022) [15]**	402 (201/arm)	CHA_2_DS_2_-VASc 4.7 ± 1.5	3.1 ± 0.9	Watchman or Amulet vs. DOAC (95% apixaban)	Median follow-up 3.5 yr (1354 patient-years). Primary composite (cardioembolism, CV death, clinically relevant bleeding, procedure/device complications): sHR 0.81 (95% CI 0.56–1.18); *p* = 0.27; *p* for non-inferiority = 0.006. Annualized rates 8.6% vs. 11.9%. Non-procedural clinically relevant bleeding: sHR 0.55 (95% CI 0.31–0.97); *p* = 0.039.	Small sample with wide confidence intervals; not powered for individual components. Single country, 10 centers. Composite is asymmetric—procedural complications can occur only in the device arm. Device-related thrombus and peridevice leak data incomplete (COVID-19).
**OPTION (2024) [12]**	1600 (1:1)	CHA_2_DS_2_-VASc 3.2	1.1	Watchman FLX vs. OAC (95% DOAC) after AF ablation	LAAO performed 90–180 days after ablation in ~60%, concomitantly in ~40%. Efficacy (all-cause death, stroke, SE at 36 mo): 5.3% vs. 5.8%; *p* < 0.001 for non-inferiority. Safety (non-procedural ISTH major or CRNM bleeding): 8.5% vs. 18.1%; *p* < 0.001 for superiority (HR 0.44; 95% CI 0.33–0.59).	All-cause rather than cardiovascular mortality avoids adjudication bias but incorporates deaths the intervention cannot modify, diluting between-group differences and making non-inferiority easier to meet; comparison with trials using cardiovascular death should allow for this. Post-ablation, low-bleeding-risk cohort limits generalizability to unselected high-risk AF populations.
**CHAMPION-AF (2026) [13]**	3000 (1:1)	CHA_2_DS_2_-VASc 3.5	1.3	Watchman FLX vs. DOAC (first-line strategy)	Efficacy (CV death, stroke, SE at 3 yr): 5.7% vs. 4.8%; difference 0.9 percentage points (95% CI −0.8 to 2.6), within the 4.8-point margin; *p* < 0.001 for non-inferiority. CV death identical (2.7% vs. 2.7%). Ischemic stroke numerically higher with the device (3.2% vs. 2.0%). Safety (non-procedural bleeding): 10.9% vs. 19.0%; HR 0.55 (95% CI 0.45–0.67); *p* < 0.001 for superiority. ISTH major bleeding alone did not differ (5.1% vs. 6.4%).	Bleeding was assessed as a separate safety endpoint and therefore does not contribute to, and cannot sustain, the efficacy non-inferiority result. Non-inferiority rested on low absolute event rates against a permissive margin: applied to the observed comparator rate of 4.8%, the 4.8-point margin admits a relative risk of 2.0, and the upper bound of the observed hazard ratio (1.20; 95% CI 0.87–1.66) does not exclude a substantial relative increase in events. Hemorrhagic stroke was identical in the two arms (0.4%), so the numerical excess within the composite is ischemic. Low bleeding risk at baseline (HAS-BLED 1.3) constrains the achievable absolute bleeding benefit. Prespecified 5-year endpoint (ischemic stroke or SE) is pending.
**CLOSURE-AF (2025 presented; 2026 published) [16]**	912 randomized (888 analyzed)	CHA_2_DS_2_-VASc 5.2 ± 1.5	3.0 ± 0.9	LAAO (Amulet or Watchman FLX) vs. physician-directed best medical care (85% received a DOAC)	Mean age 77.9 ± 7.1 yr; median follow-up 3 yr. Primary composite (stroke, SE, major bleeding BARC ≥ 3, CV or unexplained death): 16.8 vs. 13.3 events per 100 patient-years; *p* = 0.44 for non-inferiority—not met; adjusted HR 1.28 (95% CI 1.01–1.62) favoring medical care. Stroke rates similar (2.6 vs. 2.7). No reduction in major bleeding despite avoidance of long-term anticoagulation. Periprocedural complications 5.7%; serious adverse events 82.5% vs. 77.4%.	In a population at very high combined ischemic and bleeding risk, the early procedural burden offsets the long-term benefit; frailty was not an enrolment criterion. The absence of any bleeding reduction undermines the principal rationale for LAAO in precisely the population where it is most often considered. Single country, 42 centers.

BARC, Bleeding Academic Research Consortium; CHA_2_DS_2_-VASc, congestive heart failure, hypertension, age ≥ 75 years, diabetes, prior stroke or transient ischemic attack, vascular disease, age 65–74 years, female sex; CHADS_2_, congestive heart failure, hypertension, age ≥ 75 years, diabetes, prior stroke or transient ischemic attack; CI, confidence interval; CrI, credible interval; CRNM, clinically relevant non-major; CV, cardiovascular; DOAC, direct oral anticoagulant; HAS-BLED, hypertension, abnormal renal or liver function, prior stroke, bleeding history, labile INR, elderly, drugs or alcohol; HR, hazard ratio; ISTH, International Society on Thrombosis and Haemostasis; LAAO, left atrial appendage occlusion; NR, not reported; OAC, oral anticoagulation; SE, systemic embolism; sHR, subdistribution hazard ratio.

**Table 2:** Antithrombotic regimens after percutaneous LAAO: trial protocols versus real-world practice.

Source	Setting (n)	Antithrombotic Regimen	Key Observation
**PROTECT-AF/PREVAIL [9,10,11]**	RCT (707/407)	Warfarin + aspirin for 45 days; de-escalation to aspirin + clopidogrel until 6 months if peridevice leak < 5 mm; aspirin indefinitely	Regimen chosen empirically, never tested against an alternative; became the basis of the original device labeling
**CHAMPION-AF [13]**	RCT (3000)	DOAC + aspirin, DOAC alone, or DAPT for 3 months, followed by antiplatelet monotherapy	Three regimens permitted within a single trial, with no randomized comparison between them
**Freeman et al., NCDR LAAO Registry [28]**	Real-world, USA (31,994)	Warfarin + aspirin 36.9%; DOAC + aspirin 20.8%; warfarin alone 13.5%; DOAC alone 12.3%; DAPT 5.0%	Only 12.2% received the complete post-procedural protocol studied in the pivotal trials
**Reinhardt et al., NCDR, Watchman FLX era [29]**	Real-world, USA (53,878)	DOAC + aspirin 48.3%; DOAC alone 22.6%; DAPT 8.1%; warfarin + aspirin 7.7%; DOAC + P2Y12 inhibitor 4.9%	DOAC alone: lower major adverse events (HR 0.78; 95% CI 0.68–0.91) and major bleeding (HR 0.69; 95% CI 0.60–0.80) than DOAC + aspirin
**EWOLUTION [30]**	Real-world, international (1005)	At discharge: DAPT 60%; OAC 27%; SAPT 7%; no antithrombotic therapy 6%	Antiplatelet-dominant European practice, the mirror image of contemporary US practice
**Procopio et al., Italian multi-device registry [31]**	Real-world, Italy (428)	DAPT 27%; SAPT 26%; OAC 27%; other regimens 14%; none 6%	Regimens distributed almost evenly; with DAPT as reference, OAC was associated with better outcomes
**Carvalho et al. [32]**	Network meta-analysis (41 studies; 12,451)	Seven regimens compared, from no antithrombotic therapy to OAC plus antiplatelet	DOAC monotherapy ranked best for both thromboembolism and major bleeding; DAPT superior to SAPT for thromboembolic protection

CI, confidence interval; DAPT, dual antiplatelet therapy; DOAC, direct oral anticoagulant; HR, hazard ratio; OAC, oral anticoagulation; RCT, randomized controlled trial; SAPT, single antiplatelet therapy.

## Data Availability

No new data were created or analyzed in this study. Data sharing is not applicable to this article.
